# Imaging Features of Mandibular Ramus Osteoma on Computed Tomography: A Case Report With Literature Analysis

**DOI:** 10.1155/crid/1874709

**Published:** 2026-04-20

**Authors:** Emanuele Gattuso, Antonio Lo Casto, Enzo Maria Giuseppe Cumbo

**Affiliations:** ^1^ Department of Biomedicine, Neuroscience and Advanced Diagnostics (BiND), University Hospital of Palermo, Palermo, Italy, unipa.it; ^2^ Department of Precision Medicine in Medical, Surgical and Critical Care (Me.Pre.C.C.), University of Palermo, Palermo, Italy, unipa.it

**Keywords:** ascending ramus, bone tumor, CT, mandible, osteoma

## Abstract

Osteomas are benign, mesenchymal, and slow‐growing lesions that arise from the surface of the bone and consist primarily of lamellar/cortical‐type bone. They are characterized by the proliferation of compact or cancellous bone. The most common locations for osteoma to originate are the mandible and paranasal sinuses. In the mandible, the body, angle, and condyle are more common sites for osteoma than the ramus, more rarely involved. A rare case of an asymptomatic mandibular osteoma, arising from the right mandibular ramus, in a 47‐year‐old woman is described. The osteoma was incidentally discovered on the lingual surface of the ramus, extending between the internal and external pterygoid muscles into the right masticator space during a CT for oncologic follow‐up. Multiplanar and 3D reformations were acquired by a 128‐row CT device. The osteoma showed no dimensional growth during the last 2 years, and no further symptoms occurred, so the surgical procedure was not required. A comprehensive literature review of mandibular osteoma cases, classifying them according to various criteria, including the size of the lesion, the age, and gender of patients, is also presented.

## 1. Introduction

Osteoma is a benign, well‐defined, ivory‐like, and slow‐growing tumor that arises from the bone surface and is characterized by the proliferation of compact or cancellous bone. Classification of osteoma is based on location and growth pattern. According to the site, osteoma is classified as central (arising centripetally from the endosteum), peripheral (defined by centrifugal growth from the periosteum), or extraskeletal (growing within soft tissues). It is typically a slow‐growing and asymptomatic condition, frequently manifesting as an incidental finding. In the skull, the most common locations for osteoma to originate are the paranasal sinuses. In a minority of cases, osteomas may cause pain, obstruct the paranasal sinuses, or result in local swelling. In such instances, radical excision may be an appropriate treatment option [1].

Enostosis (bone island) should not be considered a medullary form of osteoma. Rather, enostosis represents the endosteal counterpart of exostosis and is regarded as a hamartomatous focus of compact cortical bone located within cancellous bone. Unlike osteoma, enostosis lacks true neoplastic behavior and typically remains stable over time. Multiple enostoses are characteristic of osteopoikilosis, whereas multiple osteomas may raise suspicion for Gardner syndrome. However, numerous dense bone islands in the jaws—particularly when multiple or extensive—may occasionally represent early skeletal manifestations associated with Gardner syndrome, sometimes preceding craniofacial osteomas and intestinal polyposis. Recognizing this potential overlap is clinically relevant, as extensive jawbone islands or coexisting osteomas should prompt careful clinical evaluation and appropriate surveillance. Computed tomography (CT) density measurements can be used to differentiate bone island from sclerotic metastases, using a cutoff of > 885 Hounsfield units [2].

A rare case of an osteoma of the mandibular ramus, extending into the masticator space, incidentally found during a CT, is described. A comprehensive literature review of mandibular osteoma cases, classifying them according to various criteria, including the size of the lesion, the age, and gender of patients, is also presented.

## 2. Case Report

A 47‐year‐old woman presented for a follow‐up CT for breast cancer. The patient had no symptoms, no difficulty opening the mouth or chewing, and no history of trauma to the right side of the mandible.

The examination was performed using a 128‐row CT scanner with the following acquisition parameters: tube voltage 120 kVp, automatic tube current modulation, slice thickness 2.5 mm, and image reconstruction using a soft tissue kernel. Images were reviewed using both standard soft tissue and dedicated bone window settings. Multiplanar and three‐dimensional reformations were subsequently generated.

**Figure 1:**
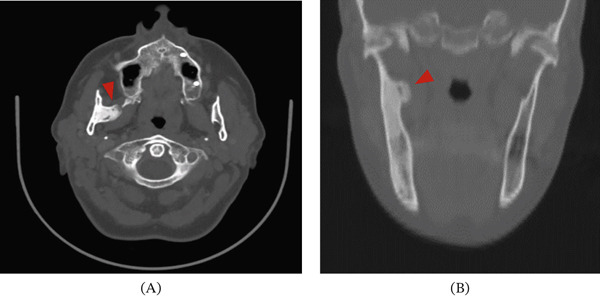
(A) Axial and (B) coronal CT images showing a mushroom‐like bone lesion with lobulated margins arising from the lingual surface of the right mandibular ramus. The lesion is located anterior to the mandibular foramen (arrowhead). The lesion extends up to the external pterygoid lamina and between the internal and external pterygoid muscles into the right masticator space.

**Figure 2:**
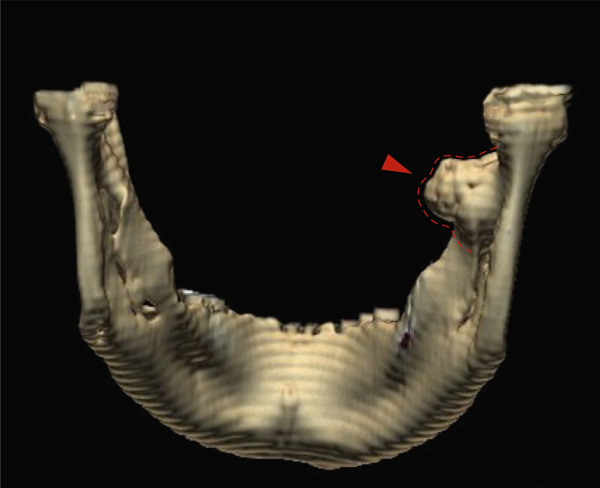
3D CT reformation of the mandible, showing the osteoma in the right ramus (arrowhead and dotted line).

The head and neck CT scan revealed a dense and homogeneous lesion, 1.8 × 1.3 cm in size, growing from the periosteum at the origin of the right mandibular ramus. The lesion was well circumscribed with lobulated margins and a mushroom‐like shape. It was attached to the lingual surface of the mandible and located anterior to the mandibular foramen, extending between the internal and external pterygoid muscles into the masticator space, pointing toward the lateral pterygoid plate (Figures 1 and 2).

Quantitative attenuation measurements were obtained, showing a mean attenuation value of approximately 1100 Hounsfield units, consistent with compact cortical bone.

The patient had undergone two additional follow‐up CT scans in the last 2 years, and the lesion showed no variations either for the CT aspect or size.

Since the lesion has remained asymptomatic and radiologically stable over a 2‐year follow‐up, no surgical procedure was planned. No histopathological specimen was obtained due to conservative management without excision. Nevertheless, its well‐circumscribed, homogeneous hyperdense appearance on CT, cortical continuity with the mandibular ramus, and lack of aggressive features were highly characteristic of a compact peripheral osteoma.

Given the patient′s history of breast cancer, osteoblastic metastasis was carefully considered in the differential diagnosis. However, this possibility was excluded based on several imaging and clinical features, including the lesion′s pedunculated morphology with cortical continuity, homogeneous compact bone attenuation, absence of contrast enhancement, lack of additional skeletal lesions, and radiological stability over a 2‐year follow‐up period. Furthermore, there was no evidence of active oncological disease or systemic progression at the time of imaging.

Other differential diagnoses included bony exostoses, ossifying fibroma, and fibrous dysplasia of the bone. However, the clinical and radiographic findings were consistent with an osteoma.

## 3. Discussion

Osteoma of the jaw is a relatively rare lesion characterized by the proliferation of compact or cancellous bone. Its etiology remains unknown. Some authors consider osteoma to be a congenital lesion, while others consider it a developmental anomaly triggered by trauma or infection, suggesting possible development from embryological cartilaginous/periosteum remnants [1]. Our case, however, did not have any history of trauma or infection.

Solitary peripheral osteoma is usually characterized by a very slow centrifugal growth from the periosteum, a unilateral involvement, well‐defined margins, and a mushroom‐like shape [1]. The clinical features of our case were consistent with these findings.

On CT imaging, osteoma should be differentiated from other radiopaque mandibular lesions, particularly bony exostosis, ossifying fibroma, and fibrous dysplasia. Osteoma typically appears as a well‐circumscribed, homogeneous hyperdense lesion composed of compact bone, showing no enhancement and maintaining cortical continuity with the underlying bone.

Exostosis is a bony outgrowth of reactive or developmental origin that is not considered a true neoplasm, and it usually stops growing after puberty. It resembles the compact subtype of osteoma, but is differentiated primarily by its typical location and shape. Three types of exostosis are identified according to their location: torus mandibularis, torus palatinus, and multiple exostoses of the molar region of the maxilla.

Ossifying fibroma (also known as cemento‐ossifying fibroma) is an encapsulated, benign neoplasm consisting of fibrous tissue that contains various amounts of irregular bony trabeculae. It usually develops in the mandible next to the root of the teeth or in the periapical region. Like osteoma, ossifying fibroma is generally an asymptomatic lesion; however, a progressive increase in size may eventually cause swelling of the mandible. On CT imaging, a mature ossifying fibroma usually appears as a well‐circumscribed, expansile lesion surrounded by a rim of less‐ossified tissue.

Fibrous dysplasia of the bone is a developmental benign medullary fibro‐osseous process characterized by the failure to form mature lamellar bone. Radiographic features of fibrous dysplasia differ considerably, but they usually present a “ground‐glass” appearance on CT, along with a focal cortical bone expansion. Varying degrees of enhancement are observed in both ossifying fibroma and fibrous dysplasia, unlike osteoma, which has no enhancement [3, 4].

The most common locations for osteoma are the paranasal sinuses, primarily the frontal sinus followed by the ethmoid and maxillary sinuses [1, 4]. In contrast to the mandibular ramus, which is only rarely affected, osteomas of the mandibular body, angle, and condyle are more frequently observed [1, 5]. In their review of the literature up until 2013, Ragupathy et al. found that, of the 87 documented cases of peripheral osteoma of the mandible, only 11% were located at the ramus [6].

Surgical excision of mandibular osteomas is generally recommended in cases of progressive growth, functional impairment (such as trismus, malocclusion, or limitation of mandibular movement), pain, facial asymmetry, or compression of adjacent anatomical structures. In asymptomatic patients with stable lesions, a conservative approach with periodic radiological follow‐up is considered appropriate.

Twenty‐seven cases of osteomas, including our case, arising from the mandibular ramus were overall found [1, 4–12] (Table 1).

**Table 1:** Mandibular ramus osteomas.

No.	Authors	Year	Age/gender	Size	Location of the lesion
1	Fordyce et al.	1953	68/F	5 × 5 × 3.8 cm	Ramus (buccal aspect)
2	MacLennan et al.	1974	31/F	NA	Ramus (buccal aspect)
3	Green et al.	1974	25/F	NA	Ramus (posterior and buccal aspect)
4	Frenkel et al.	1975	NA	NA	Ramus
5	Plezia et al.	1984	26/M	NA	Coronoid process and ramus
6	Cautley et al.	1985	NA	NA	Ramus
7	Swanson et al.	1992	22/M	6 × 4.5 cm	Ramus (posterior aspect)
8	Bodner et al.	1998	16/F	4 × 2.5 × 2 cm	Ramus (lingual aspect)
9	Longo et al.	2001	74/M	NA	Ramus (buccal aspect)
10	Sugiyama et al.	2001	51/F	1 × 0.7 cm	Ramus (lingual aspect)
11	Goodger et al.	2004	36/M	NA	Ramus (lingual aspect)
12	Woldenberg et al.	2005	16/F	4 cm	Ramus
13	Cincik et al.	2006	75/F	4 × 1 cm	Ramus (buccal aspect)
14	Donohué‐Cornejo et al.	2010	11/F	3 × 3 cm	Ramus (buccal aspect) and angle
15	Alves et al.	2011	38/F	NA	Ramus (lingual aspect)
16	Kachewar et al.	2012	50/F	10.8 × 4.0 × 4.6 cm	Ramus (buccal and lingual aspect), angle, and inferior border
17	Soni et al.	2012	60/M	5 × 3 cm	Ramus (lingual aspect), angle, and inferior border of the body
18	Shetty et al.	2014	16/F	5 × 4 cm	Ramus and angle
19	Gawande et al.	2015	45/M	5 × 4 cm	Ramus (lingual and buccal aspect), angle, and inferior border of the body
20	Sadeghi et al.	2015	53/M	9.5 × 8 cm	Ramus (lingual aspect), angle, and body
21	Ata‐Ali et al.	2016	67/F	NA	Ramus (posterior and buccal aspect)
22	Osawa et al.	2018	57/F	2.1 × 1.3 × 1.3 cm	Ramus (buccal aspect)
23	Demircan et al.	2020	17/M	4.5 × 3.5 × 3 cm	Ramus (posterior) and condyle
24	Beltrà et al.	2021	68/M	4.5 × 3.3 × 5 cm	Ramus (buccal and lingual aspect)
25	Tilaveridis et al.	2022	47/F	2.2 × 2 × 1.2 cm	Ramus (buccal aspect)
26	Fernando and Accurso	NA	52/F	2.5 × 2.5 cm	Ramus (lingual aspect)
27	Present case	2024	47/F	1.8 × 1.3 cm	Ramus (lingual aspect)

Of the 27 osteomas reviewed, 12 were located on the buccal cortical bone of the mandibular ramus, 11 on the lingual cortical bone (as in our case), and only 4 on the posterior aspect. In five cases, the exact location of the osteomas along the ramus was not mentioned. In our review, we also included cases of giant osteomas, which in the majority of instances involved multiple portions of the mandible.

Demographic data were available for 25 of the 27 cases reviewed. Based on these reports, the mean age was 42.72 years (range: 11–75 years), with a female‐to‐male ratio of 1.77:1 (16 females and 9 males). In the remaining two cases, patient sex was not specified. The largest diameter of osteomas ranged between 1 and 10.8 cm.

The osteoma of our case is notable for its proximity to the lamina of the external pterygoid process, yet it remains asymptomatic. A lesion with the same characteristics as ours has only been reported in the cases described by Longo et al. and Fernando and Accurso [1, 9].

In contrast, Bodner et al. reported a case characterized by trismus and deviation of the mandible due to a large peripheral osteoma extending into the infratemporal fossa, while Alves et al. described a painless, immobile swelling of the posterior vestibule without limitation of mouth opening [10, 13].

## 4. Conclusion

In summary, due to its rarity in the literature, we present this case as a reminder of the features of this rare lesion that could be incidentally discovered on CT imaging, particularly when the lesion does not present any symptoms, and the differences from other similar lesions of the mandible.

## Funding

No funding was received for this manuscript.

## Conflicts of Interest

The authors declare no conflicts of interest.

## Data Availability

The data that support the findings of this study are available from the corresponding author upon reasonable request.
